# Methyl salicylate 2-*O*-*β*-D-lactoside, a novel salicylic acid analogue, acts as an anti-inflammatory agent on microglia and astrocytes

**DOI:** 10.1186/1742-2094-8-98

**Published:** 2011-08-11

**Authors:** Xi Lan, Rui Liu, Lan Sun, Tiantai Zhang, Guanhua Du

**Affiliations:** 1 Beijing Key Laboratory of Drug Target and Screening Research, Institute of Materia Medica, Chinese Academy of Medical Sciences & Peking Union Medical College, No.1 Xiannongtan Street, Xicheng District, Beijing 100050, P. R. China

## Abstract

**Background:**

Neuroinflammation has been known to play a critical role in the pathogenesis of Alzheimer's disease (AD). Activation of microglia and astrocytes is a characteristic of brain inflammation. Epidemiological studies have shown that long-term use of non-steroidal anti-inflammatory drugs (NSAIDs) delays the onset of AD and suppresses its progression. Methyl salicylate-2-*O*-*β*-D-lactoside (DL0309) is a new molecule chemically related to salicylic acid. The present study aimed to evaluate the anti-inflammatory effects of DL0309.

**Findings:**

Our studies show that DL0309 significantly inhibits lipopolysaccharide (LPS)-induced release of the pro-inflammatory cytokines IL-6, IL-1β, and TNF-α; and the expression of the inflammation-related proteins iNOS, COX-1, and COX-2 by microglia and astrocytes. At a concentration of 10 μM, DL0309 prominently inhibited LPS-induced activation of NF-κB in glial cells by blocking phosphorylation of IKK and p65, and by blocking IκB degradation.

**Conclusions:**

We demonstrate here for the first time that DL0309 exerts anti-inflammatory effects in glial cells by suppressing different pro-inflammatory cytokines and iNOS/NO. Furthermore, it also regulates the NF-κB signaling pathway by blocking IKK and p65 activation and IκB degradation. DL0309 also acts as a non-selective COX inhibitor in glial cells. These studies suggest that DL0309 may be effective in the treatment of neuroinflammatory disorders, including AD.

## Findings

Alzheimer's disease (AD) is a progressive neurodegenerative disorder of the elderly characterized by global deficits in cognition ranging from loss of memory to impaired judgment. It has been hypothesized that early microglial activation in AD delays disease progression by promoting clearance of beta amyloid peptide (Aβ) before formation of senile plaques [1-3]. Microglia are antigen-presenting cells that, upon activation, are capable of phagocytosis and the production of various pro-inflammatory molecules such as nitric oxide (NO) and interleukin-1β (IL-1β) [4]. These molecules are able to destroy pathogens, but can also induce toxicity in neurons, which are compromised in AD. Furthermore, in aged human brain, many microglia are dystrophic, showing morphological features indicative of senescence such as fragmented cytoplasmic processes [5]. Like microglia, chronically activated astrocytes are believed to contribute to AD through production of NO and of various pro-inflammatory cytokines and chemokines. Apart from this, astrocytes become activated around plaques to take up Aβ and neuronal debris [6]. Not only that, activated astrocytes are also involved in plaque formation. Therefore, agents that block the activation of microglia and astrocytes may be effective in the treatment of AD.

A recent study showed that the administration of non-steroidal anti-inflammatory drugs (NSAIDs) could delay progression of AD, most likely because of their ability to reduce microglial activation and cytokine release. The presence of inflammatory processes in AD brains suggests that anti-inflammatory agents like ibuprofen may be beneficial in this disease [7]. NSAIDs have been well studied, both in vitro and in vivo, and have been observed to ameliorate inflammation related to Aβ deposition in AD. Several in *vitro *studies have shown that NSAIDs like aspirin might have anti-aggregation activity for Aβ by blocking the NF-κB signaling pathway [8].

**Figure 1:**
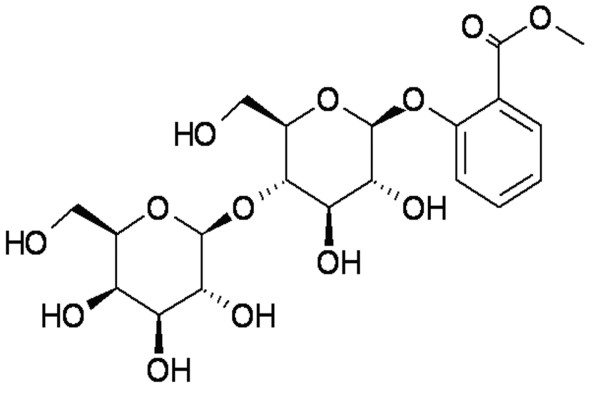
**Chemical structure of compound DL0309 (methyl salicylate 2-*O*-*β*-D-lactoside)**.

Methyl salicylate 2-*O*-*β*-_D_-lactoside (DL0309, Figure 1) was isolated from *Gaultheria yunnanensis (FRANCH) )REHDER (G yunnanensis)*, which is a traditional Chinese herbal medicine. *G yunnanensis *is widely used for the treatment of rheumatoid arthritis, swelling, and pain [9]. Interestingly, DL0309 contains a chemical structure similar to salicylic acid. Therefore, it is a natural salicylic derivative, and belongs to the NSAIDs group. An anti-inflammatory effect of *Gaultheria *has been demonstrated in a croton oil-induced ear edema model in mice [10]. Therefore, we investigated the capacity of DL0309 to suppress the production of pro-inflammatory cytokines (IL-6, IL-1β, and TNF-α) and the expression of inflammation-related proteins (iNOS, COX-1, and COX-2) in LPS-activated microglia and astrocytes, and we explored the association of these effects with activation of the NF-κB pathway.

Primary rat glia cells were obtained through a modification of McCarthy and deVellis's protocol [11]. Primary cells were cultured in DMEM/F12 medium containing 10% FBS (Gibco), 1.4 M L-glutamine, 100 U/mL penicillin, and 0.1 mg/ml streptomycin; and were found to be of 95% purity as determined by immunocytochemical staining with ox42 and anti-glial fibrillary acidic protein (GFAP) antibody.

To investigate the anti-inflammatory actions of DL0309, microglia and astrocytes were incubated with DL0309 (0.1, 1.0 or 10 μM) in the presence or absence of LPS (0.5 μg/ml) for 24 h. Pro-inflammatory cytokines (IL-6, IL-1β and TNF-α) levels in the culture medium were measured by ELISA. The production of NO-derivative nitrite was determined by the Griess reaction as described previously [12].

Western blot analysis was carried out evaluating the expression of iNOS, COX-1, COX-2, and NF-κB pathway-relevant proteins such as IκB-α, total/phosphorylated IKK and NF-κB-p65. Cells were plated overnight in 100 mm dishes and pre-treated with DL0309 at concentrations of 0.1, 1.0 or 10 μM for 1 h. After exposing the cells to LPS (0.5 μg/ml) for either 10 min or 45 min, cytosolic protein extracts were prepared. COX-1 and COX-2 antibodies were obtained from Abcam (Cambridge, UK). Other antibodies were purchased from Cell Signaling Technology (Beverly, MA, USA). Western blotting results were quantified using Quantity One software (Bio-Rad).

To determine the direct inhibitory effects of DL0309 on COX-1 and COX-2 enzymatic activities, primary rat glial cells were pre-incubated with LPS (0.5 μg/ml) for 24 h. Medium was then removed, and DL0309 (0.1, 1.0 or 10 μM) was added for 1 h. Cells were treated with arachidonic acid, 30 μM, for another 20 min, and PGE_2 _levels in the medium were then measured by ELISA [13,14]. To investigate the effect of DL0309 on COX-1 enzymatic activity, cells were not treated with LPS in order to express only the COX-1 isoform [15]. Thus, measured PGE_2 _levels represent COX-1 activity alone.

At least three independent experiments were used for data analysis. All data are presented as mean ± S.E.M. Values were compared using a t-test (two groups) or one-way ANOVA with *post-hoc *Student-Newman-Keuls test (multiple comparisons).

**Figure 2:**
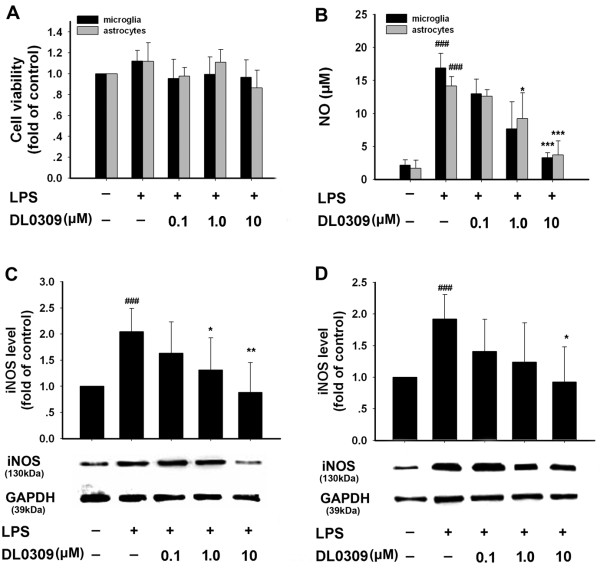
**The inhibitory effect of DL0309 on NO/iNOS induced by LPS in glial cells**. Cells were pre-treated for 1 h with the indicated concentrations (μM) of DL0309, and then stimulated by LPS (0.5 μg/ml) for 24 h. Cell viability was determined by MTT assay (A). The level of nitrite in the culture medium was determined by the Griess reaction (B). iNOS protein levels in microglia (C) and astrocytes(D) were measured by western blotting. Data represent the means ± S.E.M. of three independent experiments. ^###^p < 0.001 vs. control; *p < 0.05, **p < 0.01 and ***p < 0.001 vs. LPS-treated cultures.

Cell viability was determined by an MTT reduction assay as described previously [12]. DL0309 did not show toxicity to the cells at the concentrations examined (Figure 2A). As shown by Griess assay, incubation with LPS alone markedly increased (about 8-fold) NO production in the cells, compared to that generated in control cells; DL0309 inhibited LPS-induced NO release in microglia and astrocytes, both in a dose-dependent manner (Figure 2B). Furthermore, LPS increased the protein expression of iNOS both in microglia (Figure 2C) and astrocytes (Figure 2D), while pretreatment with DL0309 significantly decreased iNOS expression at a concentration of 10 μM. These results demonstrate that DL0309 inhibits NO release, at least in part by suppressing iNOS expression.

**Figure 3:**
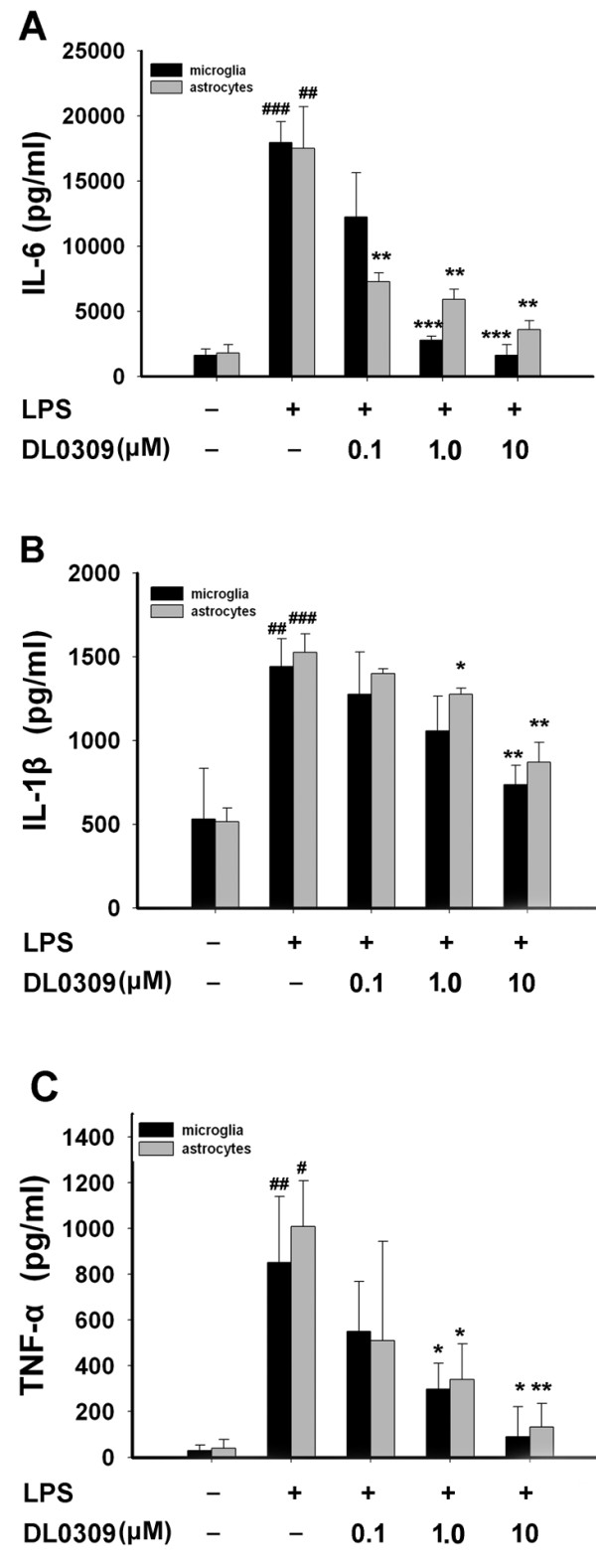
**DL0309 inhibits LPS-induced cytokine release in glial cells**. Cells were pre-treated for 1 h with the indicated concentrations of DL0309, and then stimulated by LPS (0.5 μg/ml) for 24 h. IL-6 (A), IL-1β (B), and TNF-α (C) levels were measured in the culture medium by ELISA. Data represent the means ± S.E.M. of three independent experiments. ^#^p < 0.05, ^##^p < 0.01 and^###^p < 0.001 vs. control; *p < 0.05, **p < 0.01 and ***p < 0.001 vs. LPS-treated cultures.

Neuroinflammation, represented by activated microglia and astrocytes, is a prominent pathological feature that contributes to neurodegeneration in AD. In AD brain, activated microglia release a variety of neurotoxic compounds and pro-inflammatory mediators, including IL-6, IL-1β and TNF-α. As shown in Figure 3, IL-6 (A), IL-1β (B) and TNF-α (C) levels were increased in culture medium of LPS-stimulated glial cells. Our results show that DL0309 significantly inhibits production of these proinflammatory cytokines, which may modulate AD.

**Figure 4:**
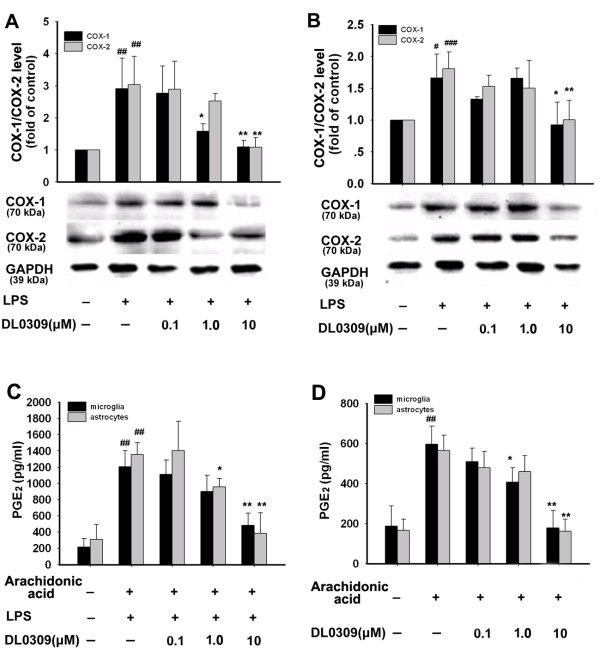
**DL0309 inhibits COX-1/2 expression and enzymatic activity in LPS- induced glial cells**. Microglia (A) and astrocytes (B) were pre-treated for 1 h with the indicated concentrations of DL0309, and continuously incubated with LPS (0.5 μg/ml) for 24 h. Levels of COX-1 and COX-1 were measured by western blotting. To measure total COX activity (COX-1/2) (C), cells were stimulated with LPS (0.5 μg/ml) for 24 h. After changing the medium, cells were treated with various concentrations of DL0309 for 1 h. Cells were then treated with arachidonic acid, 30 μM, for another 20 min, and PGE_2 _levels in the medium were then measured by ELISA. For the COX-1 activity assay (D), cells were pre-treated with various concentrations of DL0309 for 1 h. After 30 μM of arachidonic acid was added for 20 min, PGE_2 _levels in the cell medium were measured. Data represent the means ± S.E.M. of three independent experiments. ^#^p < 0.05, ^##^p < 0.01 and ^###^p < 0.001 vs. control; *p < 0.05 and **p < 0.01 vs. LPS-treated cultures.

Sustained up-regulation of pro-inflammatory mediators such as COX-1 and COX-2 in microglia and astrocytes contributes to the progressive character of AD. LPS treatment significantly increased protein expression of COX-1 and COX-2 in both microglia (Figure 4A) and astrocytes (Figure 4B). Pre-treatment with DL0309 reduced this protein expression in a dose-dependent manner in microglia. Additionally, DL0309 also directly inhibited total COX (COX-1/2, Figure 4C) and COX-1 (Figure 4D) activity in a dose-dependent manner. These results show that DL0309 acts as a non-selective COX inhibitor both in microglia and in astrocytes. These studies suggest that DL0309 may be effective in the treatment of these disorders.

**Figure 5:**
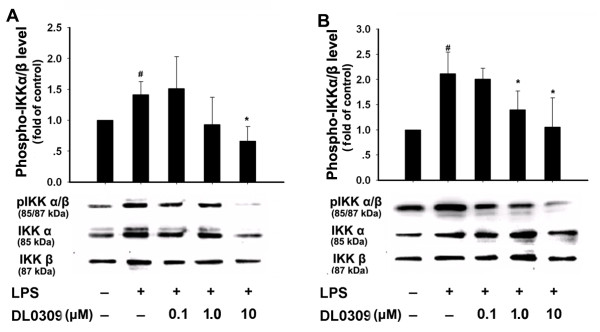
**DL0309 decreases phosphorylated IKK levels in LPS-activated glial cells**. Microglia (A) and astrocytes (B) were pre-treated for 1 h with the indicated concentrations of DL0309. LPS (0.5 μg/ml) was added and, 10 min later, proteins were isolated and the levels of phosphorylated IKKα/β, IKKα and IKKβ were measured by western blotting. Data represent the means ± S.E.M. of three independent experiments. ^#^p < 0.05 vs. control; *p < 0.05 vs. LPS-treated cultures.

**Figure 6:**
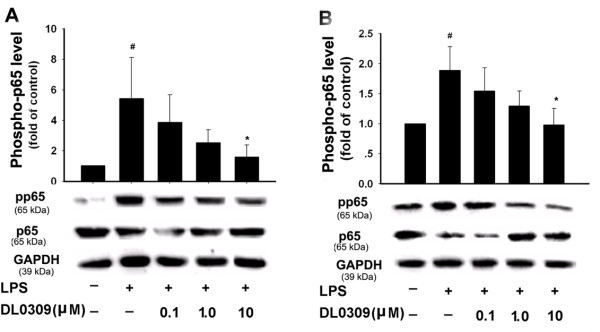
**DL0309 reduces phosphorylated NF-κB-p65 levels in LPS-activated glial cells**. Microglia (A) and astrocytes (B) were pre-treated for 1 h with the indicated concentrations of DL0309. LPS (0.5 μg/ml) was added and, 10 min later, proteins were isolated and the levels of phosphorylated NF-κB-p65 and total p65 were measured by western blotting. Data represent the means ± S.E.M. of three independent experiments. ^#^p < 0.05 vs. control; *p < 0.05 vs. LPS-treated cultures.

**Figure 7:**
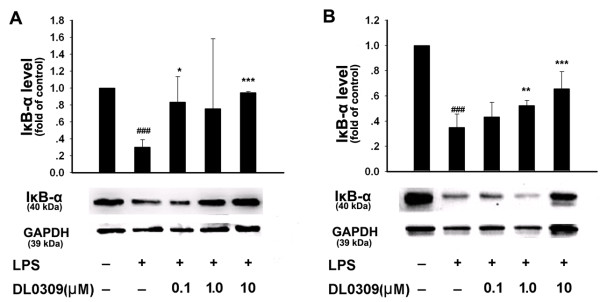
**DL0309 blocks degradation of IκB by LPS-activated glial cells**. Microglia (A) and astrocytes (B) were pre-treated for 1 h with the indicated concentrations of DL0309. LPS (0.5 μg/ml) was added and, 45 min later, proteins were isolated and levels of IκB-α were measured by western blotting. Data represent the means ± S.E.M. of three independent experiments. ^###^p < 0.001 vs. control; *p < 0.05, **p < 0.01 and ***p < 0.001 vs. LPS-treated cultures.

NF-κB is known as an important regulator of various genes involved in the production of many pro-inflammatory cytokines and enzymes related to the inflammatory process. Activation of NF-κB is critical for the expression of various cytokines, iNOS, COX-1 and COX-2 in microglia in response to LPS [16]. Normally NF-κB remains inactivated by an inhibitory protein, IκB. Once activated, NF-κB enters the nucleus to increase transcription of different inflammatory mediators. The phosphorylation of IκB is regulated by IKK. Thus, we studied the effects of DL0309 on NF-κB activation. LPS strongly increased phosphorylated IKK (Figure 5) and NF-κB-p65 (Figure 6), while simultaneously decreasing IκB expression (Figure 7) in primary microglia (A) and astrocytes (B). Our studies demonstrate that DL0309 regulates the NF-κB pathway by suppressing LPS induction of pIKK and pNF-κB-p65 activity in glial cells. We further demonstrated that DL0309 blocks LPS-induced degradation of IκB, which blocks the nuclear translocation of NF-κB.

In recent years, a number of mechanisms have been proposed to account for the protective effects of aspirin [17,18], ibuprofen [19] and other anti-inflammatory agents in AD. Studies have shown various degrees (risk reductions of up to 50%) of benefit from the use of NSAIDs on onset of disease and on dementia, with increased duration of NSAIDs use having increased protective effect against AD [20]. The best characterized action of these anti-inflammatory agents is to suppress neuroinflammation, primarily through their ability to inhibit COX, leading to reduced biosynthesis of pro-inflammatory molecules in glial cells. Several studies have shown that the onset of AD may be apparently suppressed or delayed by mixed COX-1 and COX-2 inhibitors [21].

In summary, we demonstrate that DL0309 is capable of acting as a non-selective inhibitor of COX-1 and COX-2. DL0309 also regulates the NF-κB signaling pathway not only by blocking degradation of IκB, but also by restraining pIKK and pNF-κB-p65 activity. Therefore, this agent can suppress proteins that are regulated by the NF-κB pathway, including iNOS, NO and the cytokines IL-1β, IL-6 and TNF-α. These studies suggest that DL0309 may be an effective agent in the treatment of neuroinflammatory disorders, including AD.

## Competing interests

The authors declare that they have no competing interests.

## Authors' contributions

TZ and GD directed the work, contributed to design the study, reviewed the data and wrote the manuscript; XL performed cell culture, western blot analysis, ELISA assay and NO measurements; RL and LS helped in performing NO measurements. All authors read and approved the final manuscript.
